# Supplementary material for: Preparation and Complex Characterisation of Stabilised Gold Nanoparticles: Biodistribution and Application for High Resolution In Vivo Imaging
Source: Pharmaceuticals (Basel). 2024 Nov 3;17(11):1479. doi: 10.3390/ph17111479 (PMC11597195; doi:10.3390/ph17111479)
Supplement: Supplementary file 1 [file pharmaceuticals-17-01479-s001.zip › pharmaceuticals-3143803-SI.pdf]

Supporting information

Preparation and Complex Characterisation of Stabilised Gold Nanoparticles: Biodistribution and Application for High Resolution In Vivo Imaging

Jaroslav Turánek [1,2,3,*], Pavlína Turánek Knötigová [1], Pavel Kulich [4], Radim Skoupý [5], Kamila Hrubanová [5], Naděžda Vaškovicová [6], Ladislav Fekete [7], Antonín Kaňa [8], Robert Mikulík [1] and Milan Raška [2]

[1] Neurology Department, The International Clinical Research Center ICRC of St. Anne's University Hospital in Brno, Pekařská 53, 656 91 Brno, Czech Republic; knotigova.p@seznam.cz (P.T.K.); robert.mikulik@fnusa.cz (R.M.)
[2] Department of Immunology, Faculty of Medicine and Dentistry, Palacky University Olomouc, Hněvotínská 3, 775 15 Olomouc, Czech Republic; milan.raska@upol.cz
[3] Institute of Clinical Immunology and Allergology, University Hospital Hradec Kralove and Faculty of Medicine in Hradec Kralove, Charles University, Sokolská 581, 50005 Hradec Kralove, Czech Republic
[4] Veterinary Research Institute, v.v.i., Hudcova 296/70, 621 00 Brno, Czech Republic; kulich.pavel@seznam.cz
[5] Institute of Scientific Instruments, v.v.i., AS CR, Královopolská 147, 612 00 Brno, Czech Republic; ras@isibrno.cz (R.S.);
[6] Department of Histology and Embryology, Faculty of Medicine, Masaryk University, Kamenice 753/5, 62500 Brno, Czech Republic; nadezda.vaskovicova@med.muni.cz
[7] Institute of Physics, Czech Academy of Sciences, Na Slovance 2, 18200 Prague 8, Czech Republic; fekete@fzu.cz (L.F.)
[8] University of Chemistry and Technology, Prague, Technická 5, 166 28 Praha 6, Czech Republic; antonin.kana@vscht.cz
* Correspondence: turanek@seznam.cz

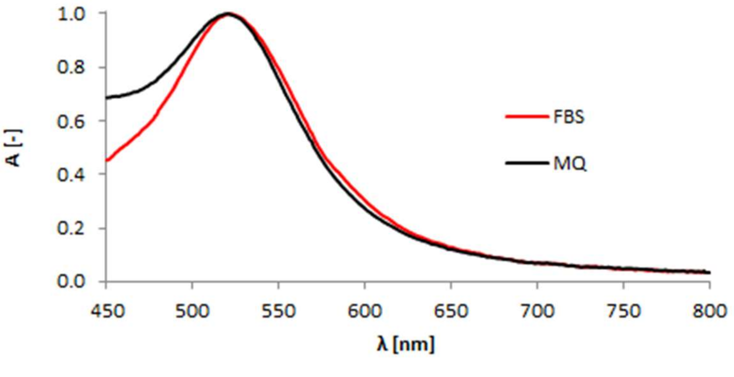

A) Difference of normalised UV-Vis spectra of PEGylated AuNPs in FBS and Mili-Q water

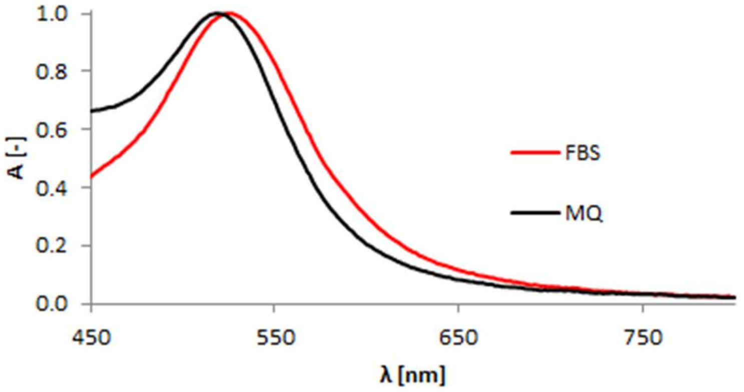

B) Difference of normalised UV-Vis spectra of citrate stabilised AuNPs in FBS and Mili-Q water

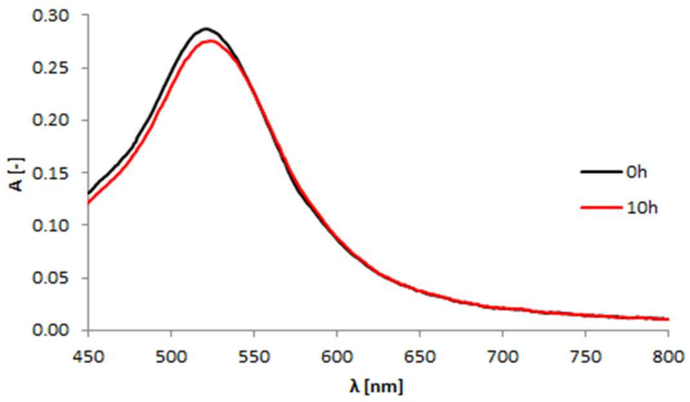

C) UV-Vis spectra of PEGylated AuNPs in FBS immediately and after 10 hours

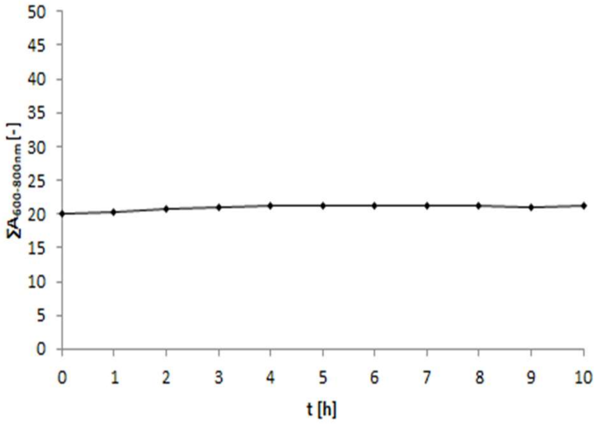

D) Suma of absorbance of PEGylated AuNPs in the region of 600 - 800 nm typical for aggregates during 10 hours in FBS sera

Figure S1. Stability of AuNP in FBS analysed by UV-Vis spectroscopy

A

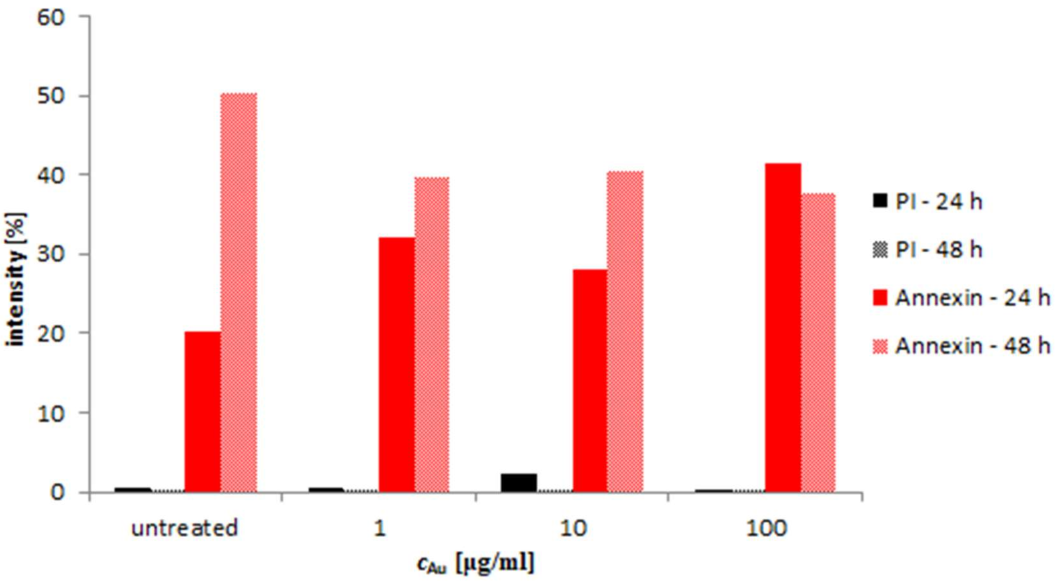

B

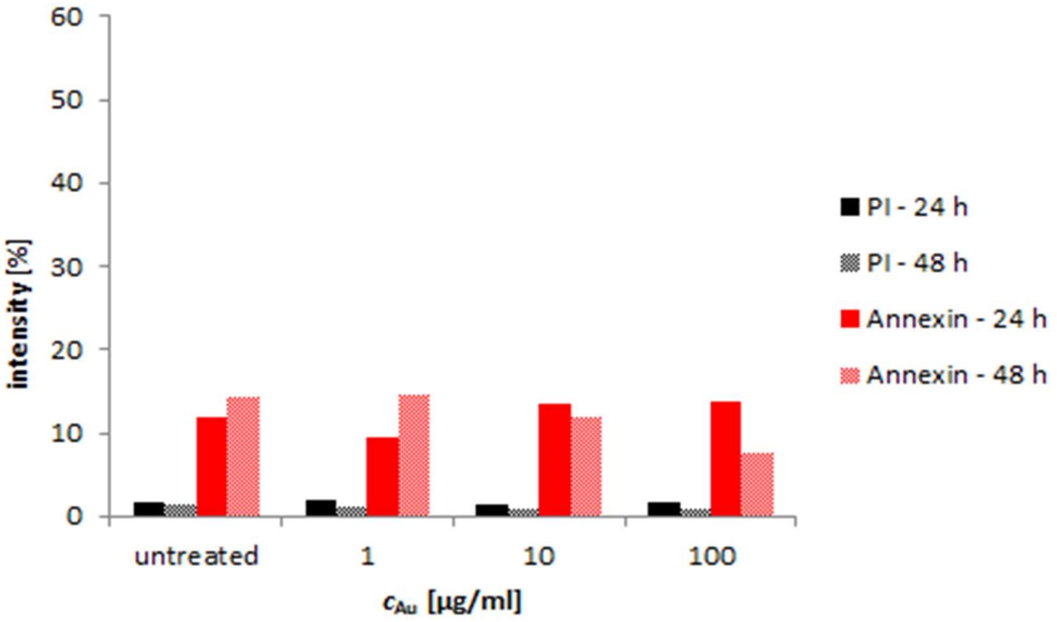

Figure S2: Cell viability of untreated and treated cells with PEGylated AuNPs after 24 and 48 hours, a) B16F1 cell line b) THP-1 cell line

Viability measurement - non adherent THP1 cells and adherent murine skin cells B16F1 were used for viability testing. We followed the experimental procedure given by the manufacturer (Tali™ Apoptosis Kit - Annexin V Alexa Fluor™ 488 & Propidium Iodide, Life Technologies Corporation) which could discriminate apoptotic from necrotic and live cells.

The cells were incubated with the concentration 100, 10 and 1 μg/ml of mPEG Au nanoparticles for 24 and 48 hrs. Negative control cells were incubating in the absence of the apoptosis inducing agent, because of removal of cells autofluorescence, we additionally prepared a second negative control of unstained cells. After exposition of drugs the cells were rinsed, incubated with Annexin V Alexa Fluor® 488, afterwards with propidium iodide and all handling were carried out according procedure recommended by the manufacturer. For the evaluation of viability flow cytometer BD FACSymphony A1 cell analyser was used.

LIVER

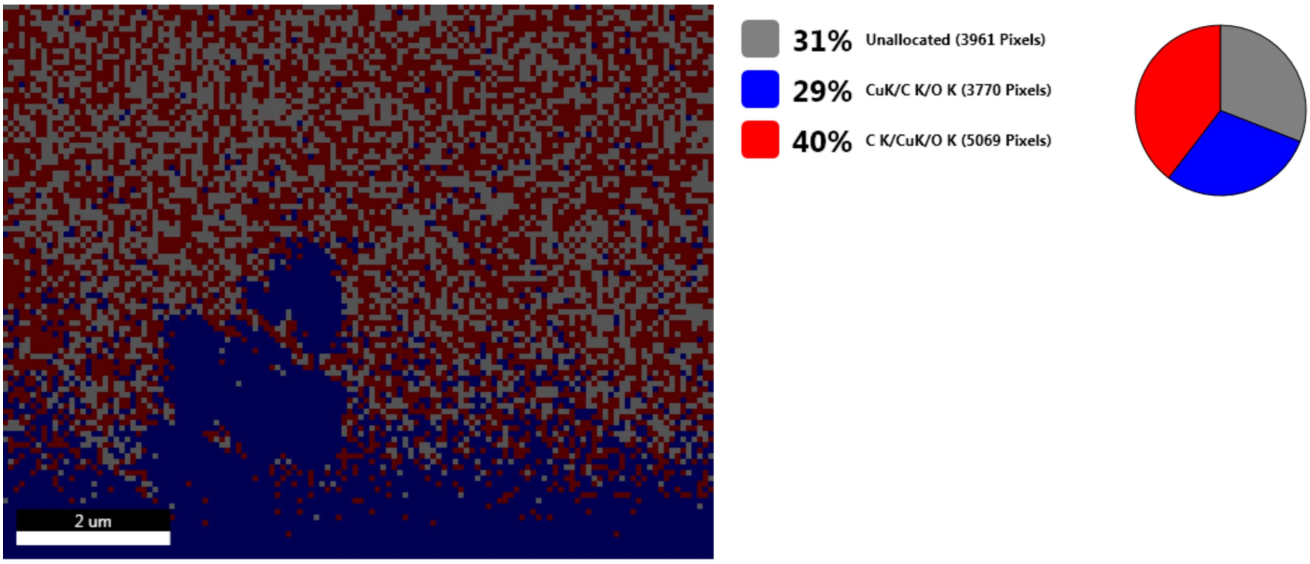
2 um
31% Unallocated (3961 Pixels)
29% CuK/C K/O K (3770 Pixels)
40% C K/CuK/O K (5069 Pixels)

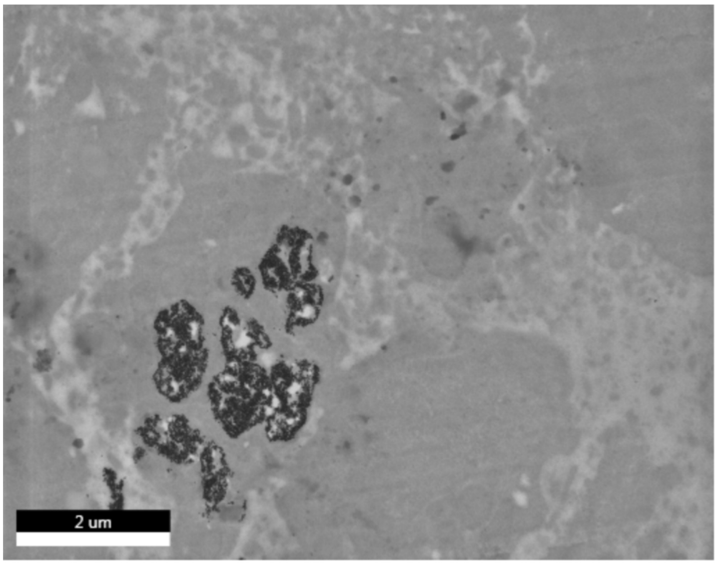
2 um

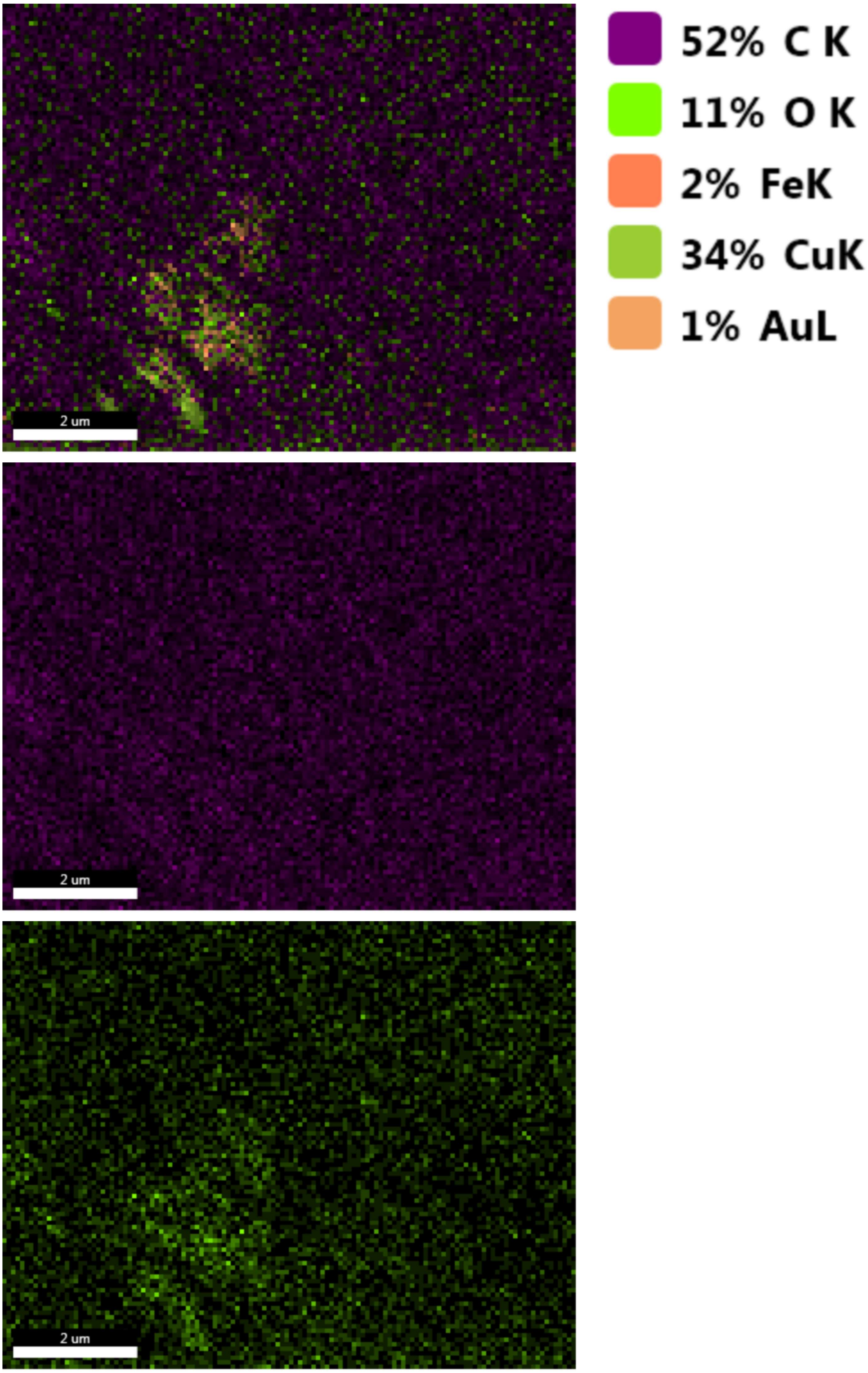
52% C K
11% O K
2% FeK
34% CuK
1% AuL
2 um
2 um
2 um

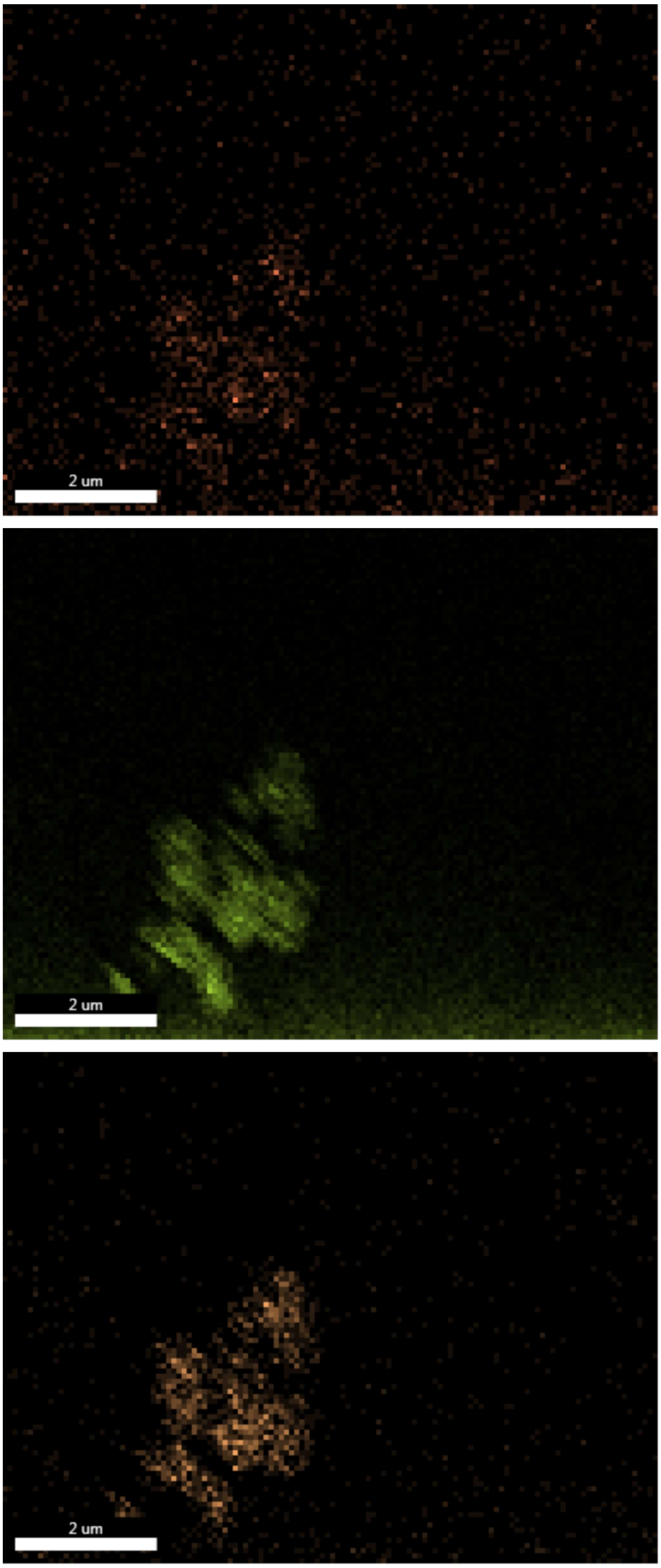
2 um
2 um
2 um

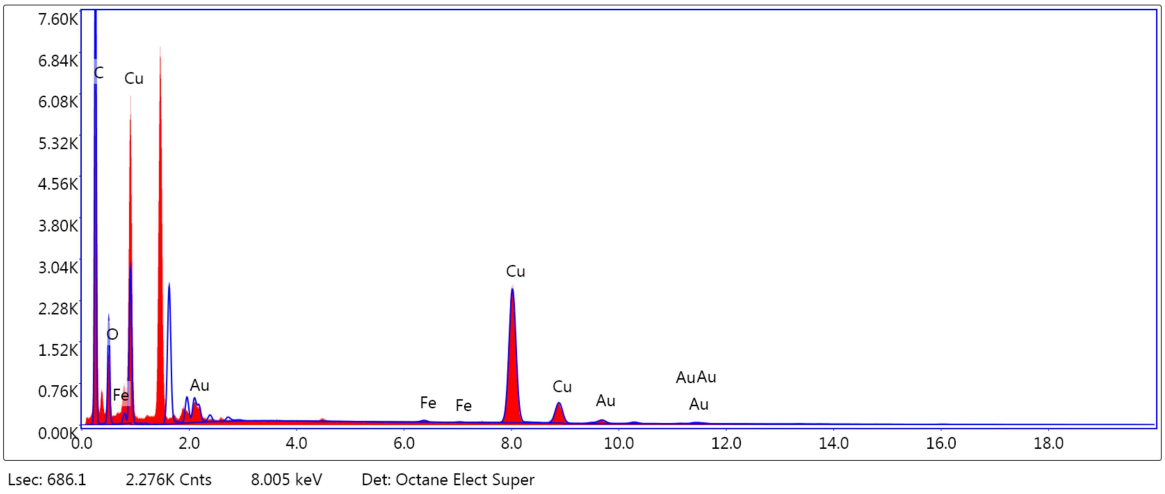

Element	Weight %	Atomic %	Net Int.	Error %	Kratio	Z	A	F
C K	64.24	77.76	112.00	6.18	0.3207	1.0390	0.4803	1.0000
O K	20.96	19.04	27.10	10.53	0.0240	1.0020	0.1141	1.0000
FeK	0.10	0.03	1.40	33.03	0.0010	0.7955	1.0467	1.2348
CuK	13.49	3.09	115.70	2.07	0.1140	0.7682	1.0396	1.0578
AuL	1.20	0.09	3.00	25.66	0.0079	0.6008	1.0965	0.9963

SPLEEN

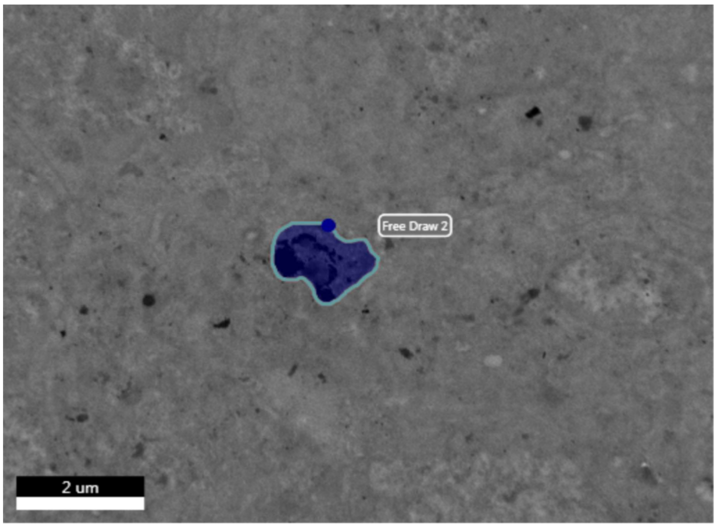

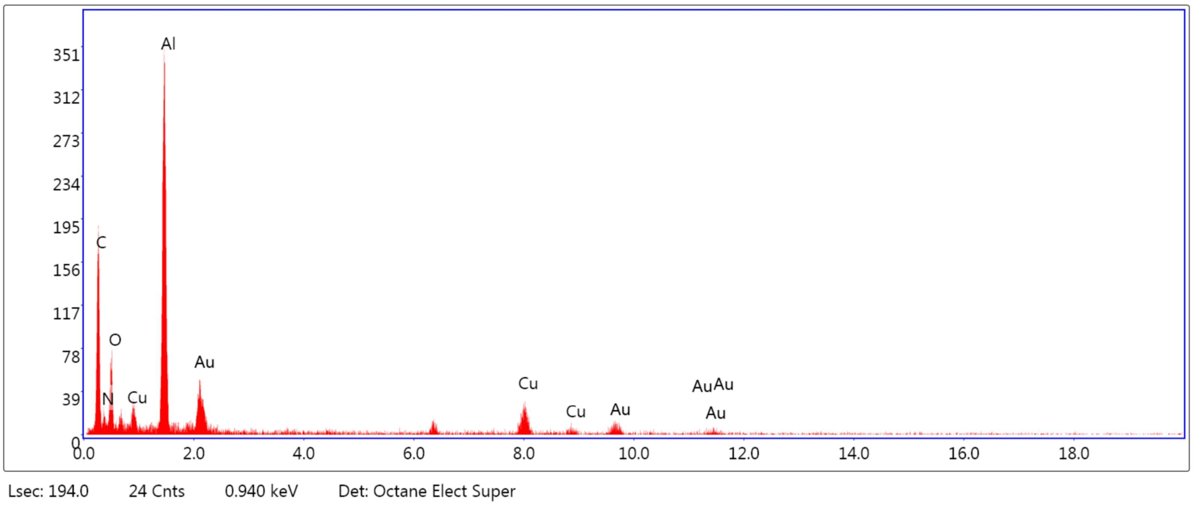

C K	47.95	58.91	10.86	9.70	0.1697	1.0394	0.3404	1.0000
N K	13.86	14.60	1.44	18.13	0.0131	1.0195	0.0927	1.0000
O K	20.80	19.19	4.51	13.69	0.0217	1.0020	0.1043	1.0000
AlK	11.90	6.51	27.25	6.97	0.0573	0.9024	0.5329	1.0011
AuM	3.07	0.23	3.60	15.17	0.0331	0.6438	1.6739	1.0019
CuK	2.42	0.56	3.76	8.60	0.0202	0.7665	1.0355	1.0536

Figure S3. Demonstration of AuNPs in histological sections from spleen and liver by TEM equipped with an EDX detector

Black spots accumulated in Kupffer cells and macrophages in liver and spleen, respectively were Au-NP, as proved by EDX detector.
